# Evaluation and Impact Reduction of Common Mode Currents on Antenna Feeders in Radiation Measurements †

**DOI:** 10.3390/s20143893

**Published:** 2020-07-13

**Authors:** Andreea Constantin, Razvan D. Tamas

**Affiliations:** 1 Department of Electronics and Telecommunications, Constanta Maritime University, 900663 Constanta, Romania; tamas@ieee.org; 2 Doctoral School of Electronics, Telecommunications and Information Technology, University Politehnica of Bucharest, 061071 Bucharest, Romania

**Keywords:** antenna radiation measurements, common mode current, distance averaging, multipath site, small antenna, loop probe, log-periodic dipole array

## Abstract

Common mode currents on antenna feeders usually occur when feeding a symmetric radiator through an asymmetric line, or when the ground plane is electrically small. Such currents may have magnitudes comparable to the feed currents and therefore have a major impact on the total radiated field. For antenna radiation measurements, both assessment and reduction of the common mode currents on antenna feeders are crucial. Techniques to discriminate antenna and feeder radiation are mainly needed for design and optimization purposes. Antenna gain measurements in a multipath site can be performed by using the distance averaging method. In this paper, we show that the distance averaging technique can be applied to reduce the effect of common mode currents for measuring the field radiated by symmetrical antennas. Two measuring configurations are proposed depending on the number of symmetry degrees of the antenna under test, and a differential approach for extracting the field created by the common mode currents was also developed. The experimental validation was performed by measuring a simple wire dipole and a log-periodic dipole array (LPDA) with a small square loop as a probe, both on the feeder side and on the feeder free side.

## 1. Introduction

The radiation originating from common mode currents has been thoroughly studied, mostly on cables attached to printed circuits boards [1,2,3,4,5]. The purpose of such studies is mainly related to the electromagnetic compatibility. When feeding symmetrical antennas or electrically small antennas through asymmetrical transmission lines (e.g., coaxial cables), common mode currents may occur on the outer conductor of the feeder. Common mode currents should normally be kept at least ten times smaller than the feed currents, in order to avoid undesirable effects. However, it has been shown [6] that common mode currents may have magnitudes comparable to the feed currents when the antenna size is comparable to the ground size or to the feed line length, and therefore have a major impact on the total radiated field.

For antenna radiation measurements, both the assessment and reduction of the common mode currents are crucial. However, there are relatively few studies focusing on the contribution to the radiation [7,8,9,10] or suppression of common mode currents [11,12]. Some authors have proposed the replacement of the coaxial cable with optical fiber for eliminating the large distortion associated with the unwanted radiation from the feed line [13].

In order to obtain accurate measurements of antenna radiation, anechoic chambers with proper shielding and absorbing material are generally employed to reduce both the interference from the external environment and the effect of the multiple propagation paths [14].

The electromagnetic field generated by common mode currents is generally measured in a single-path site such as an anechoic chamber or an open area test site (OATS).

Antenna measurements within a reverberation chamber, semi-anechoic chamber, or even a non-ideal environment will include the effect of the multipath propagation, which will result in a distortion of the measured radiation pattern [15]. In such cases, an improvement of the measuring accuracy can be achieved through removal [16,17] or compensation [18,19] of the undesired contributions.

Most of the work treating the radiation from common mode currents at a symmetrical antenna input has focused on characterizing or properly designing a balun [6,20], rather than reducing that effect by post-processing. However, using a balun in a measuring setup not only increases the cost, but would also impinge on the global frequency response.

When measuring the radiation of a symmetrical antenna such as a linear dipole, the field measured over a direction orthogonal to the antenna symmetry axis has a different magnitude on the feeder side compared to the feeder free side. The difference results from the radiation originating from the common mode currents. For a symmetrical, directive antenna (e.g., log-periodic dipole array, LPDA), common mode currents on the feeding line are commonly associated with unwanted phenomena such as the asymmetry resonance [21,22,23]. Moreover, the effect of the common mode currents on feed lines might also be of interest when designing compact LPDAs [24].

We have previously presented [25] a distance averaging method for measuring the antenna gain in a multipath site. The method basically consists of reducing the effect of the indirect paths based on the variability of their contributions to the total field compared to the direct path.

In a previous conference paper [26], we introduced a method to reduce the effect of the common mode currents for measuring the field radiated by symmetrical antennas. Our approach is based on the distance averaging technique. In this paper, we extend on our previous work as follows: (1) We propose two different approaches for this technique, depending on the number of the symmetry degrees of the antenna under test; (2) we present a distance averaging method to extract the effective area of the loop antenna that we used as a probe; and (3) we develop a differential approach for evaluating the magnetic field generated by the common mode currents on an antenna feeder.

An experimental validation was performed by measuring a simple wire dipole and a LPDA with a small square loop as a probe, both on the feeder side and on the opposite side.

## 2. Impact Reduction of the Common Mode Currents in Antenna Measurements

We considered a typical two-antenna measuring system consisting of a probe antenna (PA) and an antenna under test (AUT), respectively. As an AUT, we successively used two types of symmetrical radiators fed through coaxial cables: a two-symmetry degrees antenna (i.e., a dipole) and a one-symmetry degree antenna (i.e., a log-periodic dipole array). As a PA, we took a small, square loop antenna.

We designated as the “cable side” the field points in a direction orthogonal to the antenna, along the feed line. The “antenna side” will include field points in the same direction, but on the cable free side.

The field on the “antenna side” is entirely due to the radiation of the AUT, conversely, on the “cable side”, the field is due both to the radiation of the AUT and the cable.

**Figure 1:**
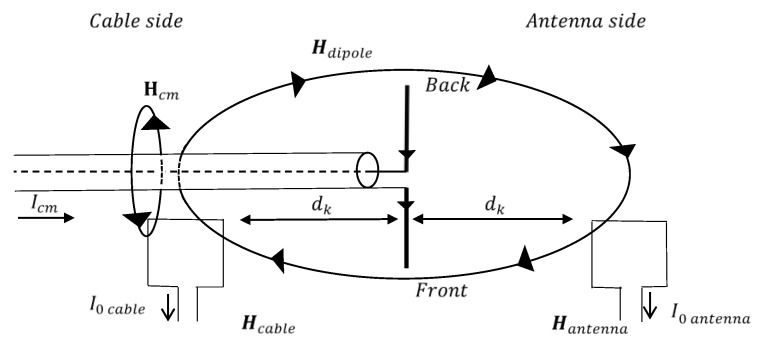
Measuring the methodology for a dipole antenna.

We propose two different measuring methodologies depending on the number of symmetry degrees of the antenna under test. When using a simple wire dipole, measurements will be performed by placing the probe on each side of the antenna (Figure 1).

**Figure 2:**
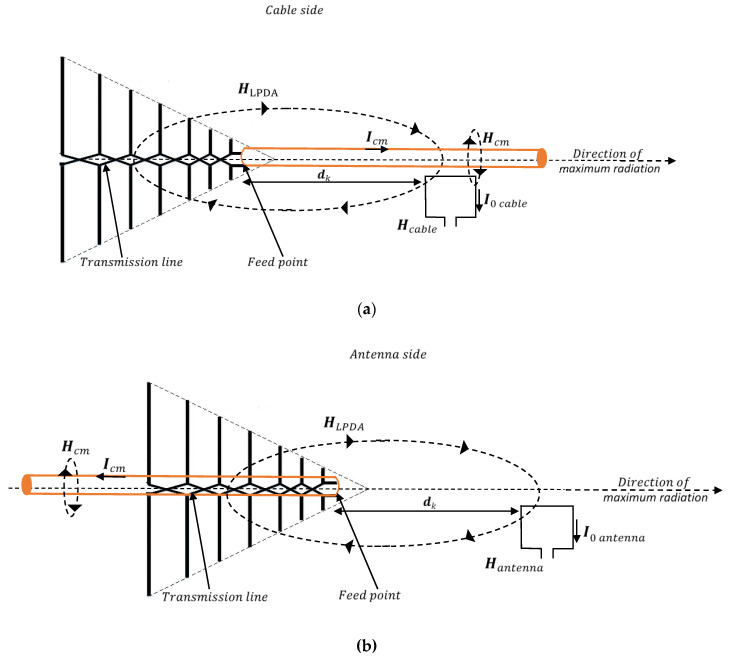
Measuring methodology for a log-periodic dipole array (LPDA): “cable side” (**a**) and “antenna side” (**b**).

When using the log-periodic dipole array as an antenna under test, measurements are performed by successively placing the coaxial cable on both sides of the feed point (Figure 2).

On the “cable side”, the PA will measure the field created by the antenna and the common mode currents on the feeder. By placing the coaxial cable in the opposite direction, the loop antenna will only measure the magnetic field generated by the log-periodic antenna.

For each type of AUT, the effect of the common mode current on the coaxial line can be assessed by subtracting the field measured at the same distance with the probe placed on the “cable side” and on the “antenna side”, respectively.

Such measurements are performed at several distances between the antenna under test and the probe, in order to apply the distance averaging approach [25].

Referring to Figure 1 and Figure 2, the magnetic field measured by the loop on the “cable side” and “antenna side” can be expressed as

(1) $$H_{cable}=H_{cm}+H_{dipole/LPDA},$$

where $H_{cm}$ is the magnetic field component generated by the common mode currents and $H_{dipole/LPDA}$ is the field component generated by the antenna, activated by the feed currents.

The contribution of the common mode current to the magnetic field can be found as

(2) $$H_{cm}=H_{cable}-H_{antenna}.$$

where

(3) $$H_{antenna}=H_{dipole/LPDA}.$$

where $I_{0\;antenna/cable}$ is the probe output current depending on the position of the loop with respect to the AUT; $A_{e}$ is the effective area of the loop; η is the free space wave impedance; and $R_{0}$ is the normalizing impedance considered as a load at the probe output.

**Figure 3:**
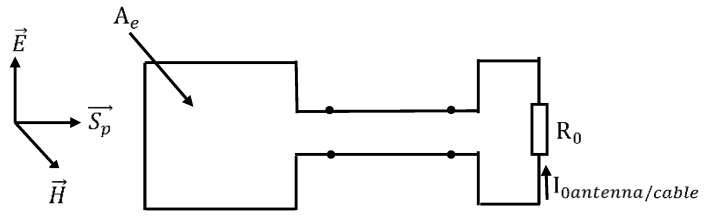
Power balance for the loop probe.

The received power can then be expressed either by integrating the incident wave power density, $S_{p}$ over the loop (Figure 3)

(4) $$P_{r}=S_{p}A_{e}=\frac{1}{2}\frac{E^{2}}{\eta }A_{e}=\frac{1}{2}\eta H_{cable/antenna}^{2}A_{e},$$

or as the power dissipated into the load at the antenna output,

(5) $$P_{r}=\frac{1}{2}R_{0}I_{0\;cable/antenna}^{2}.$$

Consequently, the magnetic field can be written as follows:

(6) $$H_{antenna/cable}=\sqrt{\frac{R_{0}I_{0\;antenna/cable}^{2}}{\eta A_{e}}.}$$

Since the circuit consisting of the probe and the AUT is terminated on the normalizing impedance at both ports, the output current can be computed as

(7) $$I_{0antenna/cable}=\frac{V_{g}\left|S_{21antenna/cable}\right|}{2R_{0}},$$

where *V_g_* is the electromotive force of the excitation at the AUT input. The contribution *S*_21*cm*_ of the common mode currents to the transfer function *S*_21*cable*_ can be derived from Equation (2),

(8) $$S_{21\;cm}=S_{21\;cable}-S_{21\;antenna}.$$

The contributions of the common mode current to the output current and to the magnetic field are given in Equations (9) and (10), respectively:

(9) $$I_{0\;cm}=\frac{V_{g}\left|S_{21\;cm}\;\right|}{2R_{0}},$$

(10) $$H_{cm}=\sqrt{\frac{R_{0}I_{0\;cm}^{2}}{\eta A_{e}}}.$$

The effective area of the loop antenna in Equation (10) should include the impedance mismatch effect both at the transmitting and receiving antennas.

**Figure 4:**
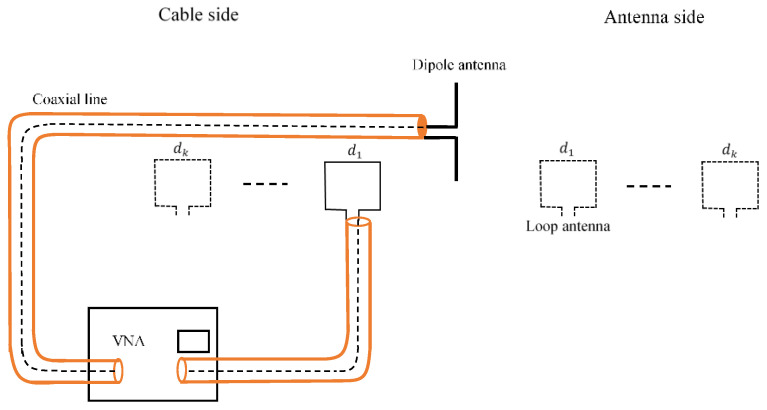
Distance averaging technique applied for reducing the effect of the common mode current on the dipole antenna radiation measurements.

**Figure 5:**
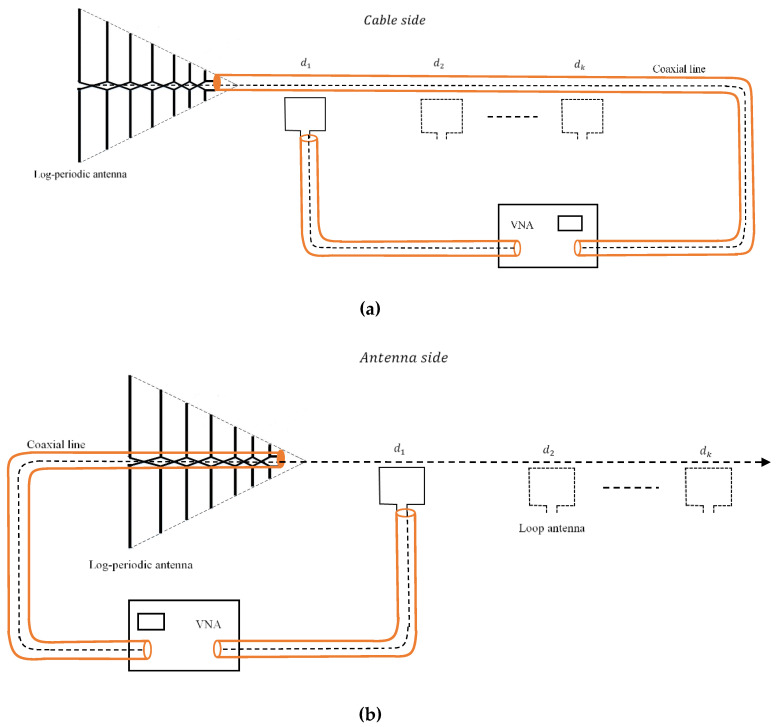
Distance averaging technique applied for reducing the effect of the common mode current on LPDA radiation measurements: “cable side” (**a**) and “antenna side” (**b**).

When transfer functions are measured at *N* different distances, an average can be computed over that dataset by compensating the effects of the propagation in terms of attenuation and delay; we have previously used such a distance averaging technique [25] with the aim to reduce the effects of the multipath propagation for antenna gain measurements. As the field corresponding to indirect propagation paths, common mode currents also have a distance variant distribution. The application of the distance averaging (Figure 4 for dipole antenna and Figure 5 for log-periodic dipole array) might therefore significantly reduce the impact of the common mode current on the antenna radiation measurements.

The average transfer function can be computed from the transfer functions $S_{21\;cm}^{d_{k}}$ measured at each distance $d_{k}$ between antennas,

(11) $${\bar{S}}_{21\;cm}=\overset{N}{\underset{k=1}{\sum }}\frac{d_{k}}{d_{0}}\exp\left(jk_{0}d_{k}\right)S_{21\;cm}^{d_{k}},$$

where $d_{0}$ is the reference distance (set at 1 m) and $k_{0}$ is the free space wavenumber. Average figures can then be derived both for output currents and magnetic field components; such figures can be defined for the “antenna” and ”cable side”, and for the common mode contribution, respectively:

(12) $${\bar{I}}_{0\;cm/cable/antenna}=\frac{V_{g}\left|{\bar{S}}_{21\;cm/cable/antenna}\;\right|}{2R_{0}},$$

(13) $${\bar{H}}_{cm/cable/antenna}=\sqrt{\frac{R_{0}{\bar{I}}_{0\;cm/cable/antenna}^{2}}{\eta A_{e\text{\_}cm/cable/antenna}}}.$$

The effect of the common mode currents should be reduced for the “cable side” measurements and therefore, corrected figures should be calculated,

(14) $$H_{cable\;corr}^{d_{k}}=\frac{d_{k}}{d_{0}}\exp\left(-jk_{0}d_{k}\right){\bar{H}}_{cable}.$$

Relation (14) gives the field value at a given distance by simply multiplying the result by that distance, provided that the average figure corresponds to a distance of 1 m between antennas.

## 3. Probe Antenna Calibration

**Figure 6:**
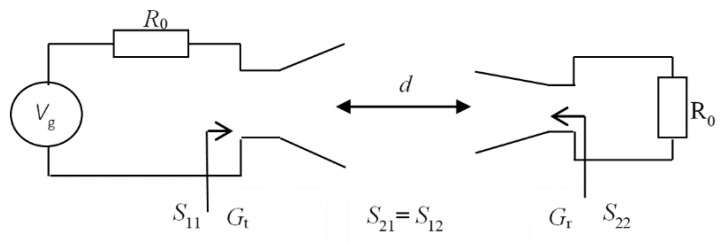
Probe calibration setup.

The gain of the loop antenna that we used as a probe can be found by characterizing the transmission between the probe and a calibrated antenna (Figure 6).

**Figure 7:**
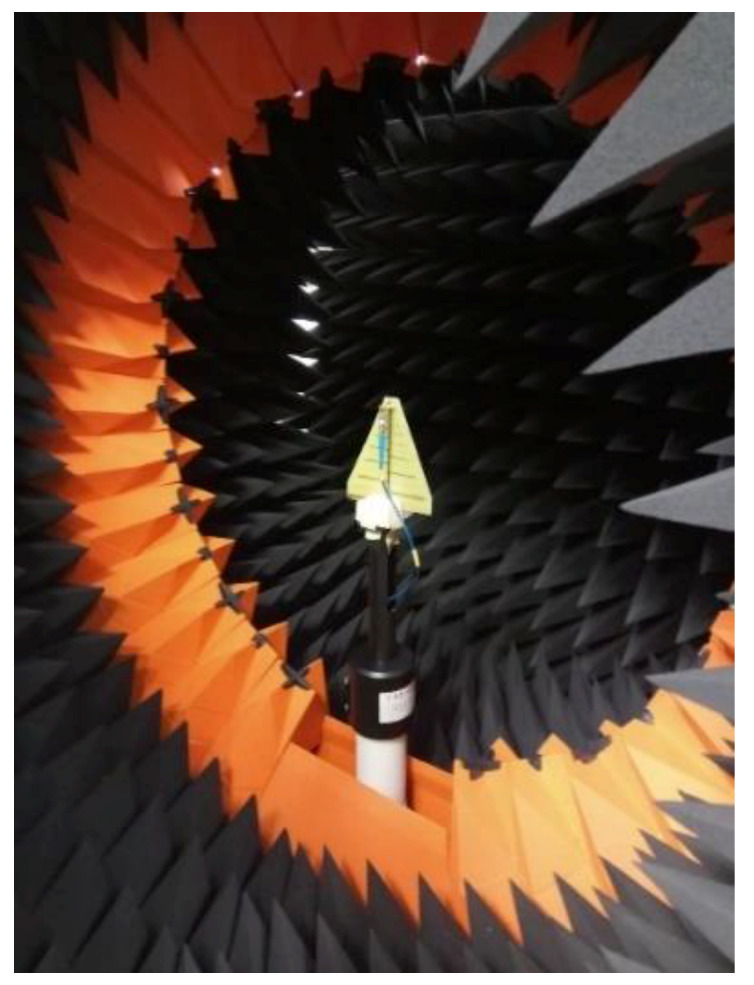
LPDA calibration.

One of the AUTs (i.e., the LPDA) has previously been calibrated inside a professional, compact range in an “antenna side” setup (Figure 7). The measuring system consists of a circular array of probe antennas placed inside an anechoic chamber, a calibrated RF generator, and a calibrated receiver, respectively. The AUT (i.e., the LPDA) was placed on a turntable. As a result, the near-field was measured on a closed surface, and the realized gain of the AUT could be accurately extracted through near-field to far-field transformations.

As a result, that AUT could itself be used as a probe for calibrating the loop when the LPDA is in an “antenna side” configuration. Furthermore, the loop calibrated as described previously will be able to measure the radiation of any other configuration (e.g., with the LPDA) in a “cable side” setup, or the dipole in an “antenna side” and “cable side” setup.

With the notations in Figure 6 and taking into account the impedance mismatch at both antennas, the gain of the receiving antenna (i.e., the AUT) can be found from the Friis formula; since part of the measurements are usually performed at the Fresnel zone ranges, a field-zone correction factor, F(f,d) should be applied on the measured results, as we have proposed in a previous paper [27]. Since the loop is used as a probe (receiving) antenna, one should characterize it through its effective area, rather than its gain, that is,

(15) $$A_{e}=\frac{4\pi r^{2}}{G_{t}}\frac{R_{0}}{R_{a2}}\frac{{\left|F\left(f,d\right)S_{21}\right|}^{2}}{{\left|1-S_{22}\right|}^{2}\left(1-{\left|S_{11}\right|}^{2}\right)}.$$

where $R_{a2}$ is the radiation resistance of the AUT and λ is the wavelength.

**Figure 8:**
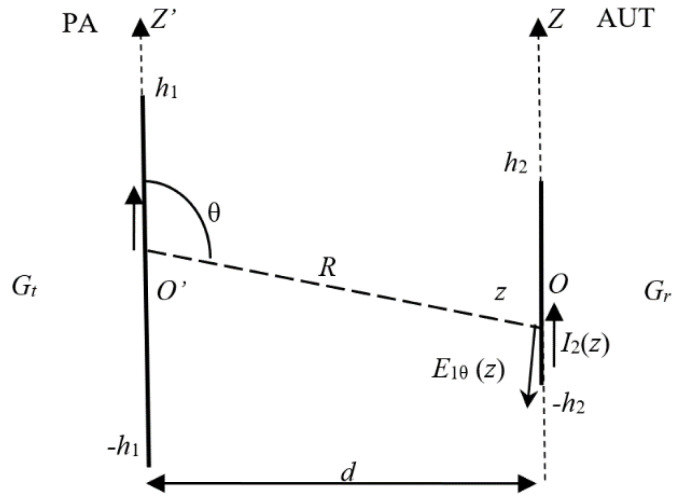
Transmission between two linear antennas.

When both antenna aperture sizes ($2h_{1}$ and $2h_{2}$ in Figure 8) are comparable to the measuring range, the lower limit of the Fraunhofer zone is found as

(16) $$d\geq \frac{8{\left(h_{1}+h_{2}\right)}^{2}}{\lambda }.$$

**Figure 9:**
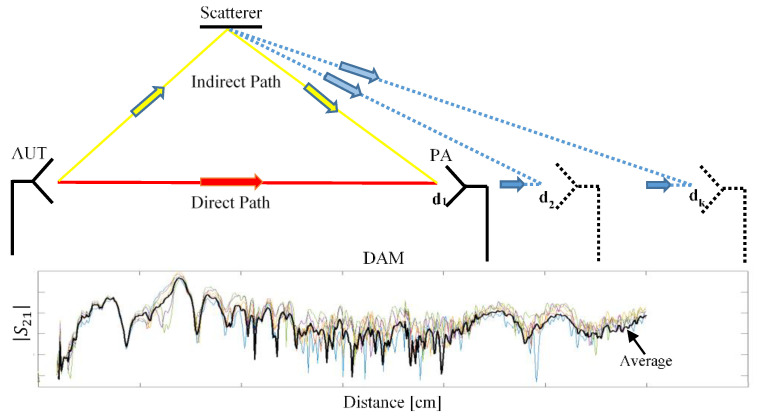
Distance averaging method.

In order to accurately evaluate the effective area of the probe antenna in a multipath environment, we used the distance averaging method [25,28]. The setup in shown in Figure 9.

The average transfer function can be expressed as in Equation (11) and the loop effective area can be found from Equation (15).

## 4. Results

**Figure 10:**
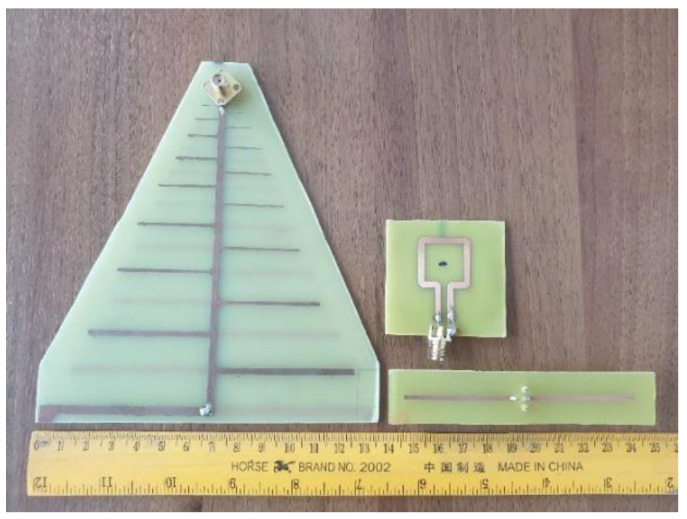
Antennas under test and probe antenna.

In order to validate our approach, we measured a dipole and a LPDA, respectively, by using a square loop probe (Figure 10). The dipole was resonating around 1.2 GHz and had a total length of 9 cm. The LPDA was designed for the frequency range 800 MHz−3 GHz and was 13 × 13 cm in size. As a probe, we used a square loop with a side length of 2 cm. Both probe and AUT were connected to a VNA for measuring the scattering parameters. The measurements were performed in a non-anechoic environment (a regular room inside a building). Measured data were then processed with a MATLAB code implementing relations (9) to (14), in order to apply our distance averaging approach, and to further extract the contribution of the common mode currents.

Since the LPDA was calibrated inside a compact range in an “antenna side” type configuration, we first extracted the effective area of the loop when placed in the same configuration. We used the distance averaging approach as the measurements were performed in an environment with multiple propagation paths.

**Figure 11:**
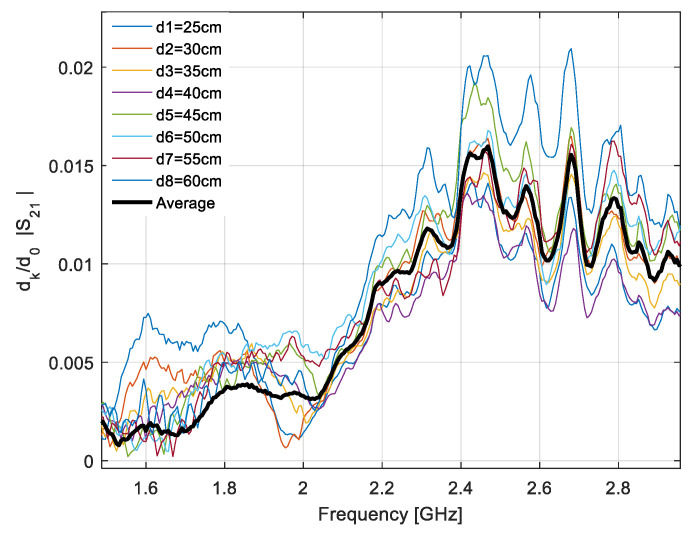
Normalized transfer functions and the average figure.

Figure 11 shows the normalized transfer functions of the antenna system measured at eight different distances ranging between 25 and 60 cm, and the average transfer function computed as in (11).

**Figure 12:**
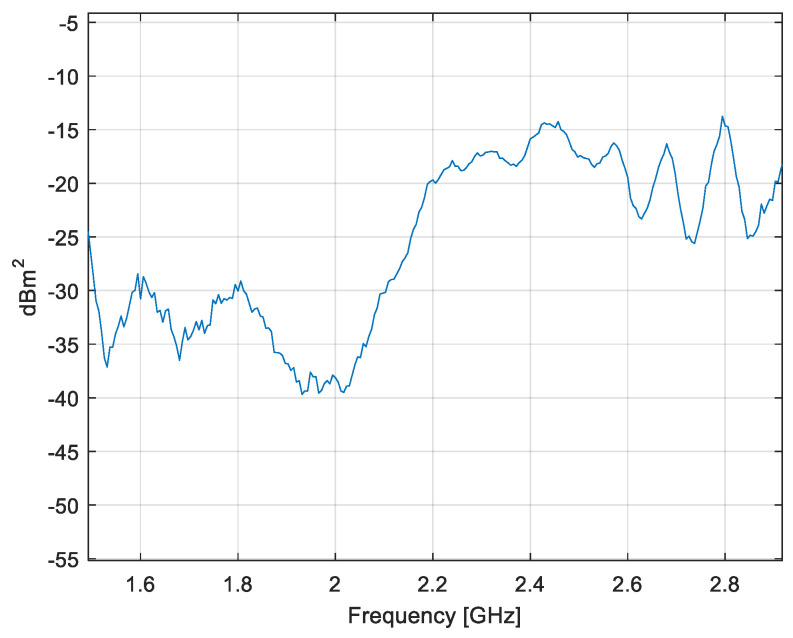
Effective area of the loop probe.

The effective area of the loop as a function of frequency is given in Figure 12.

**Figure 13:**
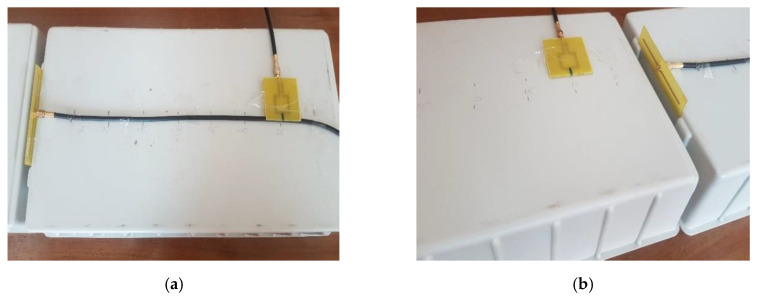
Measuring setup for a dipole antenna: “cable side” (**a**) and “antenna side” (**b**).

**Figure 14:**
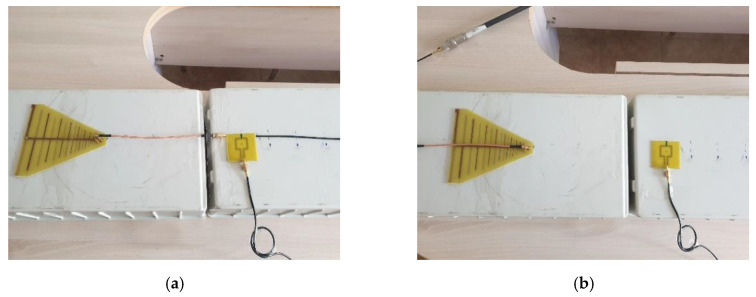
Measuring setup for a LPDA: “cable side” (**a**) and “antenna side” (**b**).

Once the loop probe was calibrated, we assessed the effect of the common mode currents by measuring the transfer functions on both the “antenna side” and ”cable side”. The setup for each AUT is presented in Figure 13 and Figure 14, respectively.

For the dipole antenna, the measurements on the “cable” and “antenna side” were performed at distances between antennas ranging from 5 to 40 cm with a distance increment of 5 cm. For the LPDA, the distance to the probe ranged between 25 and 60 cm with the same increment. All the distances corresponded at least to the Fresnel zone [27].

**Figure 15:**
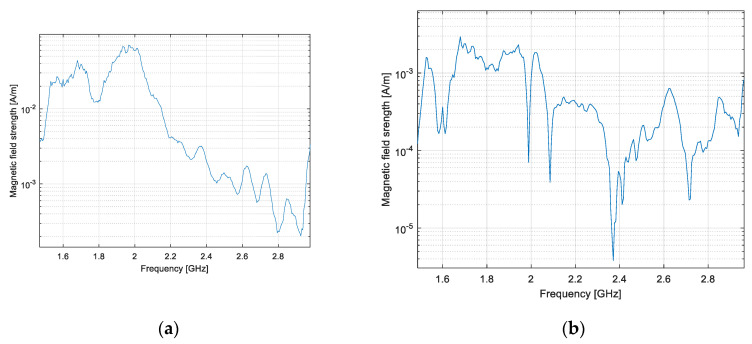
Magnetic field generated by common mode currents: dipole (**a**) and LPDA (**b**).

The measurements on both antennas were performed between 1.5 and 3 GHz, a frequency range where the loop has a good radiation efficiency. The effect of the impedance mismatch at the probe output was corrected on the measured data. The magnetic field generated by the common mode currents can be evaluated as in (3) by subtracting the results measured on the “antenna side” from those measured on the “cable side” (Figure 15).

**Figure 16:**
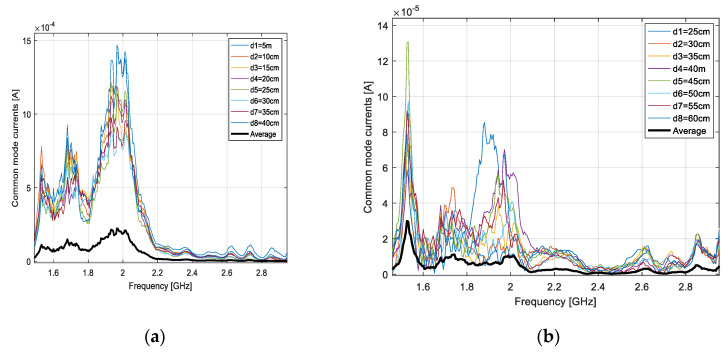
Contribution of the common mode current to the output current versus distance averaged figure: dipole (**a**) and LPDA (**b**).

In Figure 16, we show the contribution of the common mode current to the output current, as given by (9), with *S*_21_ measured at each of the eight distances between the loop and AUT. On the same diagram, we give the average figure resulting from (12) after evaluating the normalized, distance averaged transfer function as defined in (11). As Figure 16 shows, the common mode contribution to the output current can be dramatically diminished by applying the distance averaging technique.

**Figure 17:**
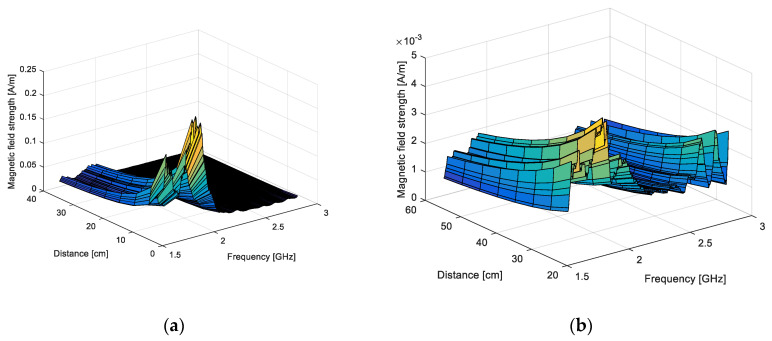
Magnetic field measured on the “cable side” after correction, as a function of distance and frequency: dipole (**a**) and LPDA (**b**).

The magnetic field on the “cable side” can be expressed by using (13), and the figure corrected with the common mode current effect by using (14). In Figure 17, we give the variation of the corrected, magnetic field strength as a function of distance and frequency.

**Figure 18:**
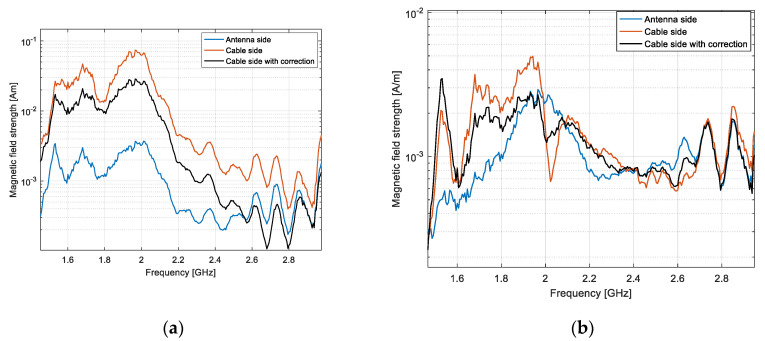
Magnetic field measured on “antenna side” and “cable side”, with and without correction: dipole antenna (**a**) and LPDA (**b**); the distance between the probe and the AUT was set at 40 cm.

Figure 18 shows a comparison between the magnetic field measured on the “cable side” at 40 cm, with and without correction of the common mode current effect, and the magnetic field on the “antenna side” at the same distance.

It appears that by applying our distance averaging technique, the corrected magnetic field magnitude on the “cable side” got closer to the magnetic field strength measured on the “antenna side”. We defined a root mean square error by taking the field strength on the cable free side as a reference. The error decreased from 71% down to 29% for the dipole and from 6.2% down to 3.1% for the LPDA.

## 5. Conclusions

In this paper, we proposed a differential approach for evaluating the magnetic field generated by the common mode currents on an antenna feeder by subtracting the magnetic field magnitude on the “antenna side” from the same figure measured on the “cable side”.

In order to extract the effective area of the loop probe, we applied a distance averaging technique derived from an approach originally developed for antenna gain measurements in a multipath site.

We also developed a distance averaging approach for correcting the field radiated by a symmetrical antenna fed through a coaxial line, with the effect of the common mode current. The common mode current has a distance variant distribution and therefore, its effect on the field measured aside the feeder can be diminished by averaging the results acquired at different distances between the probe and the antenna under test. By applying the proposed technique, a magnetic field value corresponding to a reference distance of 1 m was first derived; the actual corrected field value at the “cable side” distance was then found by multiplying the result by the distance and the corresponding phase factor.

The method was successfully validated on a symmetric wire dipole as an antenna under test and a square loop as a probe. Our distance averaging approach could also be applied to reduce the radiating effect of the common mode currents on the feed line of a LPDA. The root mean square error on the measured field magnitude was reduced by a factor of two for both antennas under test. However, common mode currents had a stronger impact for the dipole antenna since for the LPDA, we used a more balanced feeding circuit.

Several factors may impact on the accuracy of our approach, and will be investigated in a future work. First, our field zone extrapolation method might not be accurate enough for distances between the probe and AUT falling in the near-field zone. Second, a low ratio between the field generated by the common mode currents and that radiated by the antenna would make the former less discernable. Finally, one should bear in mind that the loop probe was calibrated on the “antenna side” configuration, but the probe radiation properties may change on the “cable side” due to the feeder proximity.
